# Dietary Patterns Are Associated with Blood Cell Profiles and the Molecular Composition of Platelet-Rich Plasma

**DOI:** 10.3390/nu18010163

**Published:** 2026-01-03

**Authors:** Hadrian Platzer, Alena Bork, Simone Gantz, Baraa Khamees, Maciej J. K. Simon, Sébastien Hagmann, Yannic Bangert, Babak Moradi

**Affiliations:** 1 Department of Orthopedics and Trauma Surgery, University Medical Center Schleswig-Holstein, Campus Kiel, Arnold-Heller-Straße 3, 24105 Kiel, Germany; hadrianmarius.platzer@uksh.de (H.P.);; 2 Orthopedic Research Center, Kiel University, Michaelisstr. 5, 24105 Kiel, Germany; 3 Department of Orthopedics, Heidelberg University Hospital, Schlierbacher Landstraße 200a, 69118 Heidelberg, Germanyyannic.bangert@med.uni-heidelberg.de (Y.B.)

**Keywords:** dietary pattern, inflammation, cytokine, growth factor, platelet-rich plasma, osteoarthritis, regenerative therapies

## Abstract

Background/Objectives: Platelet-rich plasma (PRP) is increasingly used in musculoskeletal medicine. Variability in PRP composition, driven by preparation- and donor-related factors, is considered a major contributor to inconsistent clinical outcomes. This study investigated whether habitual dietary patterns are associated with the cellular and molecular composition of leukocyte-poor PRP (LP-PRP). Methods: In this cross-sectional study, 75 healthy adults (25 vegans, 25 vegetarians, and 25 omnivores) who adhered to their dietary patterns for ≥6 months were enrolled. LP-PRP was prepared by a standardized protocol. Cell profiles were quantified in whole blood and LP-PRP; LP-PRP proteins (IL-6, IGF-1, HGF, and PDGF-BB) were measured by ELISA. Group differences, correlations, and multivariable regressions were performed. Results: Whole blood differed by diet with respect to total leukocytes, lymphocytes, and basophils, while platelet and erythrocyte counts did not. In LP-PRP, platelet enrichment ratios and leukocyte counts were comparable across diets. IL-6 in LP-PRP was lower in vegans vs. omnivores (*p* = 0.017); the Animal-Based Diet Score correlated positively with LP-PRP IL-6 and remained independently associated in regression (β = 0.35, *p* = 0.004). While IGF-1, HGF, and PDGF-BB did not differ between dietary groups, intake-based analyses revealed associations between specific dietary components and LP-PRP proteins; notably, the fruit and vegetable intake correlated inversely with PDGF-BB, and platelet–growth factor coupling was most pronounced among omnivores. Conclusions: Dietary patterns were associated with selected molecular components of LP-PRP—most consistently IL-6—while cell counts remain largely unchanged. However, interventional studies are needed to establish causality and determine whether dietary modification can influence clinical outcomes.

## 1. Introduction

Platelet-rich plasma (PRP) is increasingly used to treat musculoskeletal disorders, including osteoarthritis (OA). PRP is prepared by concentrating platelets from a patient’s own blood with variable leukocyte content depending on the preparation method, resulting in a cocktail enriched in growth factors (e.g., PDGF, HGF, IGF-1) and cytokines [1] that is believed to stimulate tissue regeneration and modulate inflammation when applied. However, current evidence for PRP across musculoskeletal indications is heterogeneous, and this also applies to its use in osteoarthritis [2,3,4,5], one of the most common clinical indications for PRP. In consequence, the American College of Rheumatology (ACR) and the Osteoarthritis Research Society International (OARSI) do not currently recommend the routine use of PRP for the treatment of symptomatic knee or hip OA [6].

To clarify the evidence of PRP biological activity in tissue regeneration and immunomodulation, its underlying mechanisms need to be investigated in greater detail. In this context, the variability of the PRP product itself is a major challenge: differences in preparation methods (e.g., single vs. double spin, leukocyte content, activation protocols) and donor factors (e.g., age, sex, comorbidities, medication intake) lead to heterogeneous PRP compositions [1,7,8,9,10,11,12] and are likely among the main contributors to the inconsistent results observed. This variability hampers the development of mechanistic studies and cross-study comparability and represents a major obstacle to strengthening the evidence base. It is, therefore, of critical importance to identify and investigate the factors that influence the bioactive components of PRP.

Among donor-related factors, habitual diet has emerged as an important determinant of systemic cellular and molecular profiles [13,14,15,16,17,18]. In a randomized intervention study, adherence to a Mediterranean diet, compared with a low-fat control diet, was shown to maintain platelet counts—the primary source of growth factors in PRP—within a physiological range [13]. Furthermore, its adherence has been associated with changes in systemic leukocyte counts [19], the second major cellular source of proteins in PRP, as well as significantly reduced circulating IL-6 levels [14], a potent immunomodulatory cytokine, underscoring the impact of nutrition on systemic molecular profiles and inflammation.

Because PRP is prepared directly from whole blood, these established systemic effects suggest that habitual dietary patterns may also influence the cellular and molecular composition of PRP itself, thereby potentially contributing to interindividual variability in PRP composition. However, to the best of our knowledge, the association between different dietary patterns and the cellular and molecular composition of PRP has not yet been systematically investigated. To address this gap, our study compared three distinct dietary patterns (vegan, vegetarian, and omnivore), characterized whole blood and LP-PRP cellular compositions, and quantified protein concentrations in LP-PRP lysates, combining group contrasts with intake-based correlations and regression models to investigate the association between dietary patterns and the cellular and protein profiles of PRP. Hereby, we quantified key bioactive proteins in LP-PRP—insulin-like growth factor-1 (IGF-1), hepatocyte growth factor (HGF), platelet-derived growth factor-BB (PDGF-BB), and the immunomodulatory cytokine interleukin-6 (IL-6)—which collectively play important roles in tissue regeneration and inflammation [20,21,22,23,24,25]. By establishing baseline associations in a well-controlled healthy cohort, this exploratory study aims to provide insights into how diet may modulate PRP composition and contribute to its interindividual variability.

## 2. Materials and Methods

### 2.1. Study Population—Inclusion and Exclusion Criteria

This cross-sectional observational study was conducted at the Department of Orthopedics, Heidelberg University Hospital, Germany. Healthy volunteers were recruited on a voluntary basis between 2022 and 2023 according to predefined inclusion and exclusion criteria. To minimize confounding when analyzing the associations between dietary patterns and both whole blood and LP-PRP parameters, we recruited a healthy cohort and applied comprehensive inclusion and exclusion criteria. The inclusion criteria comprised healthy adult volunteers aged 18–35 years. At the time of enrollment, none of the participants showed any clinical or laboratory evidence of acute or chronic inflammation as determined by their medical history and complete blood count, including their differential leukocyte counts.

A comprehensive set of exclusion criteria was applied to define a healthy, low-confounding cohort for analyzing the associations between dietary patterns and LP-PRP parameters: Individuals with a history of malignancy, prior chemotherapy, hematologic diseases, diabetes, other immune-related disorders, pathological obesity, or smoking were excluded. Further exclusion criteria comprised acute musculoskeletal injury, surgical procedures within the past six months, and the use of disease-modifying antirheumatic drugs (DMARDs), corticosteroids, or psychotropic agents within three months prior to study entry. In addition, an intake of non-steroidal anti-inflammatory drugs (NSAIDs) or paracetamol during the previous six weeks, as well as acetylsalicylic acid within the last two weeks before enrollment, led to exclusion from participation.

The study protocol received approval from the Ethics Committee of the University of Heidelberg (S 631/2021) on 15 September 2021. Before participation, all volunteers provided written informed consent.

### 2.2. Blood Sampling

Peripheral venous blood was obtained from the antecubital vein. For hematological assessment, samples were collected into EDTA tubes (S-Monovette^®^ EDTA K3E, 2.7 mL; Sarstedt, Nümbrecht, Germany). Additional blood (15 mL) was drawn using a commercial double-syringe device (ACP system; Arthrex, Naples, FL, USA) for the preparation of platelet-rich plasma. No activation agents or other additives were used. All specimens were processed immediately after collection, as described previously [26].

### 2.3. PRP Processing

PRP was generated using the commercial ACP double-syringe system following centrifugation at 1500 rpm (≈385× *g*) for 5 min (Horizon 24-AH, Drucker Diagnostics, Port Matilda, PA, USA), yielding approximately 5–6 mL of LP-PRP. The processing steps followed the manufacturer’s recommendations [27] and were performed consistently across all samples. No protease inhibitors were added. The samples were processed on ice and stored at −80 °C within 30 min after blood sampling to minimize protein degradation. The resulting PRP was aliquoted into 1.5 mL tubes (Eppendorf AG, Hamburg, Germany) and stored at −80 °C (Dometic UF 755 GG Ultra Low Temperature Freezer; Dometic, Solna, Sweden). To ensure platelet lysis and uniform protein release, two freeze–thaw cycles were conducted [28]. Anticoagulation before the first thaw was achieved by adding 20 µL of unfractionated heparin-sodium (25,000 I.U./5 mL; LEO Pharma A/S, Ballerup, Denmark) per 1 mL of PRP to prevent fibrin clot formation during freeze–thaw lysis and subsequent handling of the PRP. Aliquots were prepared according to downstream analysis and kept frozen until further assays were conducted.

### 2.4. Hematological Assessment

Cellular composition was determined in the whole blood and PRP samples using an automated hematology analyzer from Sysmex XN-1000 (Sysmex Corporation, Kobe, Japan) to quantify platelets, erythrocytes, and leukocytes. This procedure ensured normal baseline cell values, excluded any participant with signs of infection, and confirmed successful platelet enrichment in PRP, with a mean platelet enrichment factor of approximately 2.09 ± 0.35 across all dietary groups.

### 2.5. Protein Quantification

Protein quantification focused on LP-PRP lysates as the primary matrix of interest. After thawing the PRP samples to room temperature, they were immediately centrifuged and filtered (AcroPrep Advance 96 Well Plates, 350 µL, 3 μm glass fiber/0.2 μm Supor membrane; Pall Corporation, Port Washington, NY, USA) by centrifugation at 1400× *g* for 10 min to remove cell fragments. Protein concentrations of IL-6, IGF-1, PDGF-BB, and HGF were determined via enzyme-linked immunosorbent assays (ELISA; R&D Systems, Minneapolis, MN, USA, and Sigma-Aldrich, St. Louis, MI, USA; lot numbers: IGF-1—P310291, IL-6—P320482, PDGF-BB—0207K0180, HGF—0308K0201). All assays were performed according to the manufacturers’ instructions, ensuring reproducibility. The IL-6 assay (Quantikine^®^ High Sensitivity Human IL-6 ELISA, R&D Systems, Minneapolis, MN, USA) detects total IL-6, including both free and receptor-bound forms, as specified by the manufacturer. Thus, the measured concentrations represent the overall IL-6 levels in PRP lysates. Absorbance was measured at 450 nm using a microplate photometer (Autobio Labtec Instruments Co., Zhengzhou, China; AUTOsoft software, version 2.6.9).

### 2.6. Assessment of Dietary Patterns

A characterization of dietary patterns was assessed using items of the validated food frequency questionnaire (FFQ) that was developed for the German Health Interview and Examination Survey for Adults (DEGS) by the Robert Koch Institute (RKI) [29]. For each aggregated food group—(processed) meat, fish, non-meat animal-derived foods (e.g., milk, eggs, cheese), and fruits/vegetables—participants reported their habitual intake frequency during the last 6 months on a food-frequency questionnaire (FFQ) with a 6-point scale (1 = never; 2 = once per month or less; 3 = 2–4 times per month; 4 = 2–3 times per week; 5 = 4 times per week to daily; 6 = several times per day). Based on self-reporting, individuals were categorized into one of three dietary patterns: vegan, vegetarian, or omnivore. In accordance with the screening questionnaire, vegans were defined as individuals who consumed neither meat nor fish nor any animal-derived foods, vegetarians as individuals who abstained from meat and fish but regularly consumed non-meat animal-derived foods, and omnivores as individuals who reported a habitual intake of non-meat animal-derived foods, fish, and meat. Only individuals adhering to their respective diets (vegan, vegetarian, or omnivore) for at least 6 months prior to study enrollment were included.

An internally developed Animal-Based Diet Score was constructed as an exploratory index to quantify adherence to animal-based dietary patterns, based on the reported frequencies of (processed) meat and non-meat animal-derived food intake, with higher scores reflecting a greater intake of these foods. The score was derived from validated items of established food frequency questionnaires that are used in national health and nutrition surveys in Germany [29] and which showed internal consistency (Cronbach’s α = 0.73). To support interpretability and convergent validity, aggregated food items (processed) meat, fish, fruit, and vegetable intake, as well as the consumption of non-meat animal-derived foods, were considered separately as independent dietary variables and analyzed as separate dietary variables.

### 2.7. Statistical Analysis

The main objective of this exploratory study was to determine cytokine and growth factor concentrations in LP-PRP and to examine their associations with vegan, vegetarian, and omnivorous dietary patterns. Data are presented as the mean and standard deviation (SD) together with interquartile ranges (IQR) for continuous measures, and as counts and percentages for categorical measures. The distribution of continuous variables was assessed using the Shapiro–Wilk test.

As the continuous parameters did not follow a normal distribution, the non-parametric Kruskal–Wallis test (H test) was applied to compare demographic, hematological, and protein parameters between the three dietary patterns (vegan, vegetarian, and omnivore). Where an overall significance was reached, post hoc comparisons using Mann–Whitney U-tests with a Bonferroni adjustment were performed to adjust for multiple testing among the three dietary groups. Group differences in categorial variables (e.g., sex distribution) were assessed with the Chi-square test.

To minimize type I error inflation, we distinguished between a priori–defined primary analyses (group comparisons of LP-PRP protein concentrations and multivariable regressions of IL-6 on the Animal-Based Diet Score) and exploratory analyses (correlation analyses). For primary analyses, Bonferroni correction adjustments were applied as appropriate. Exploratory correlations were interpreted descriptively and labeled as hypothesis-generating.

Spearman’s rank correlation coefficient (ρ) was calculated in cases of non-normally distributed variables and when relationships were expected to be non-linear to evaluate the associations between hematological parameters, protein concentrations, and dietary indices (Animal-Based Diet Score, non-meat animal-derived foods, (processed) meat, fish, and fruit and vegetable consumption).

In addition, multiple linear regression analyses were performed to examine the independent association of the Animal-Based Diet Score with PRP protein concentrations (IL-6, IGF-1, HGF, and PDGF-BB), adjusting for age, sex, and BMI, as potential confounders known to affect PRP composition. Protein concentrations were log10-transformed prior to regression to approximate normality. Residual diagnostics, including Q–Q plots and standardized residuals versus predicted values, were examined to verify model assumptions, showing approximate normality and homoscedasticity.

All tests were two-tailed, and a level of *p* < 0.05 was considered statistically significant. For descriptive purposes, correlations with *p*-values between 0.05 and 0.10 were considered trend-level associations, indicating effects that approached but did not reach conventional statistical significance. For key findings, exact *p*-values are reported in the Section 3. All statistical procedures were conducted using IBM SPSS Statistics (Version 31.0.0.0 (117) IBM Corp., Armonk, NY, USA). GraphPad Prism (Version 10.0; GraphPad Software Inc., La Jolla, CA, USA) was used exclusively for data visualization and a graphical representation of the results.

## 3. Results

### 3.1. Baseline Characteristics

A total of 75 healthy participants (42 women and 33 men), evenly distributed across the three dietary groups, were included in this cross-sectional study conducted in an observational setting. The study population predominantly consisted of physically active adults from the Heidelberg metropolitan region, representing a homogeneous and healthy cohort. The mean age of the cohort was 25.92 ± 4.17 years, with a mean BMI of 22.56 ± 3.17 kg/m^2^. The sex distribution, mean age, sport activity level, and BMI were comparable between the dietary groups, with no indication of systematic demographic imbalance (Table 1).

The intake of (processed) meat and fish showed a distinct pattern; no consumption was reported among vegans and vegetarians, whereas omnivores indicated a substantially higher intake (*p* < 0.001). Non-meat animal-derived foods followed a gradient, with vegans reporting no use, and vegetarians showing intermediate intake levels, while omnivores demonstrated the highest consumption overall (*p* < 0.001). Fish consumption differed as well: both vegans and vegetarians reported no intake, while omnivores reported a higher consumption (*p* = 0.001). Fruits and vegetables consumption also differed across the three dietary patterns as expected, with vegans and vegetarians reporting a higher intake compared to omnivores (*p* = 0.001). Expected differences were confirmed, with the Animal-Based Diet Score highest in omnivores and lowest in vegans.

### 3.2. Hematological Parameters in Whole Blood and LP-PRP

In whole blood, leukocyte counts differed significantly overall between dietary groups (*p* = 0.024), with higher values in vegetarians than vegans (*p* = 0.020) (Table 2).

Likewise, lymphocyte proportions varied (overall *p* = 0.039), with a trend toward higher levels in omnivores compared to vegans (*p* = 0.055). A small, yet significant, difference was observed in basophils (overall *p* = 0.043), with significantly lower values in vegetarians compared to vegans (*p* = 0.038). No significant differences were observed for the platelet or erythrocyte counts, nor for monocyte, eosinophil, or neutrophil percentages in whole blood.

Within LP-PRP, platelet counts, leukocyte counts, and all leukocyte subtypes were comparable across dietary groups (all *p* > 0.05). Likewise, the PRP-to-whole blood platelet ratio showed no differences between dietary patterns (*p* = 0.846).

### 3.3. Protein Concentrations in LP-PRP Among Dietary Patterns

**Figure 1:**
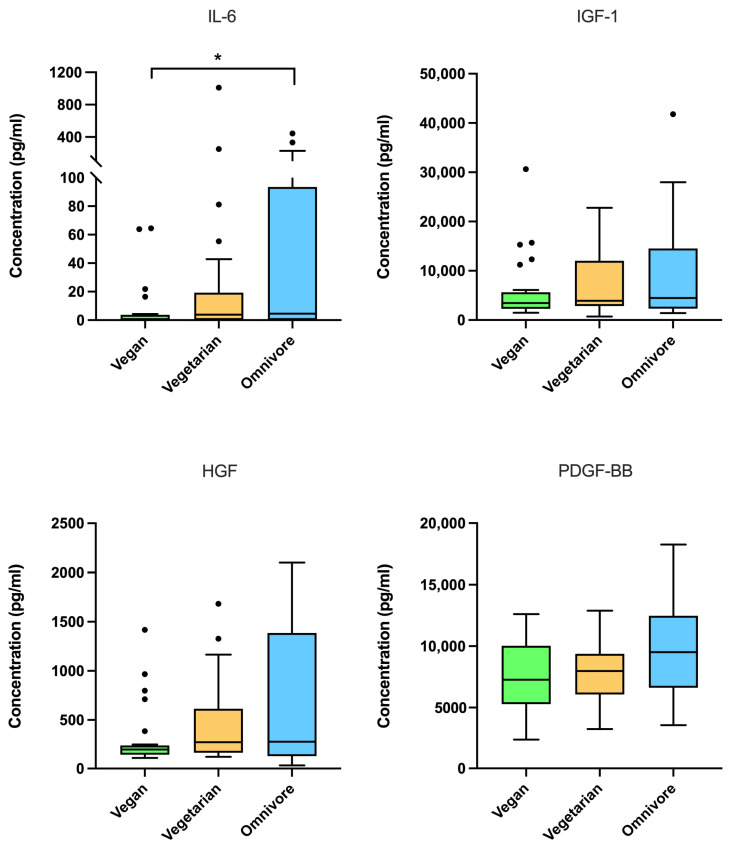
Concentrations of cytokines and growth factors in LP-PRP stratified by dietary pattern (n = 25 per dietary group). Box and whisker plots (Tukey style) show concentrations of IL-6, IGF-1, HGF, and PDGF-BB in LP-PRP obtained from participants following vegan (green), vegetarian (yellow), or omnivorous (blue) diets. Boxes represent the interquartile range (IQR) with the horizontal line indicating the median; the whiskers extend to 1.5 × IQR, and the outliers are shown as individual points. * *p* < 0.05 indicates statistical significance.

The cytokine and growth factor concentrations are visualized in Figure 1, which displays the medians, interquartile ranges, and individual data points of the outliers for each dietary group. For IL-6, the Kruskal–Wallis test indicated a statistically significant difference between the dietary groups (H(2) = 8.00, *p* = 0.018, η^2^ = 0.11), corresponding to a medium effect size. Post hoc comparisons confirmed higher IL-6 levels in omnivores compared with vegans (*p* = 0.006). After Bonferroni correction for multiple testing, this difference remained statistically significant (adjusted *p* = 0.017), whereas comparisons between vegans and vegetarians (adjusted *p* = 0.174) and between vegetarians and omnivores (adjusted *p* = 1.000) were not significant. In contrast, no significant overall between-group differences were observed for IGF-1 (H(2) = 1.81, *p* = 0.405, η^2^ = 0.02), PDGF-BB (H(2) = 4.40, *p* = 0.111, η^2^ = 0.06), or HGF (H(2) = 1.76, *p* = 0.415, η^2^ = 0.02), with effect sizes in the small to small–medium range.

### 3.4. Platelet–Protein Correlations Between Dietary Patterns

Correlational analysis between the platelet counts and protein concentrations in LP-PRP, stratified by dietary patterns in whole blood and LP-PRP, is shown in Table 3.

In whole blood, PDGF-BB correlated positively with the platelet levels in individuals following a vegan diet, whereas no significant associations were observed for IL-6, IGF-1, or HGF across the dietary groups.

In LP-PRP, more consistent relationships emerged. PDGF-BB demonstrated the strongest and most robust association, showing significant positive correlations with platelet levels across all dietary patterns. Interestingly, IL-6, IGF-1, and HGF showed positive correlations with platelet counts in PRP in the omnivorous group, whereas corresponding associations in vegans and vegetarians did not reach statistical significance.

### 3.5. Correlations of Dietary Variables and Hematological Parameters in Whole Blood

The correlation analyses indicated that the Animal-Based Diet Score was positively associated with lymphocyte proportions in whole blood (ρ = 0.320, *p* = 0.008). No significant correlations were observed with monocytes, eosinophils, basophils, or erythrocytes.

Fish consumption was also positively correlated with lymphocyte proportions (ρ = 0.279, *p* = 0.020).

A higher frequency of fruit and vegetable intake was positively associated with neutrophil proportions (ρ = 0.237, *p* = 0.042), while lymphocyte proportions in whole blood were negatively correlated on a trend level (ρ = −0.280, *p* = 0.074). Additionally, eosinophil proportions were inversely related to fruit and vegetable intake (ρ = −0.280, *p* = 0.016). No significant correlations were observed with platelet or leukocyte counts, monocytes, basophils, or erythrocyte concentrations.

### 3.6. Correlations of Dietary Variables and Hematological Parameters in LP-PRP

In LP-PRP (processed meat), the frequency of fish consumption and the Animal-Based Diet Score showed no significant association with the hematological parameters (Table 4).

Non-meat animal-derived food intake was positively correlated with leucocyte counts (ρ = 0.255, *p* = 0.032) and negatively with basophils (ρ = −0.236, *p* = 0.048). The fruit and vegetable intake was on a trend level that was positively correlated with basophils (ρ = 0.255, *p* = 0.054).

### 3.7. Correlations of Dietary Variables and Protein Concentrations in LP-PRP

Regarding protein concentrations, the Animal-Based Diet Score showed a modest positive correlation with IL-6 (ρ = 0.356, *p* = 0.003) (Table 5).

Hereby, IL-6 concentrations correlated positively with both meat consumption (ρ = 0.242, *p* = 0.045) and non-meat animal-derived food intake (ρ = 0.407, *p* < 0.001). Associations of IGF-1, PDGF-BB, and HGF, and the Animal-Based Diet Score did not reach significance. However, PDGF-BB, but not IGF-1 and HGF, showed a modest positive association with meat intake (ρ = 0.261, *p* = 0.032) and was inversely correlated with fruit and vegetable consumption (ρ = −0.340, *p* = 0.003). Fish consumption was unrelated to any of the measured proteins.

Given these bivariate relationships, multiple regression analyses were subsequently performed to assess whether dietary patterns independently predicted protein concentrations when accounting for participant characteristics.

### 3.8. Regression Analysis

**Figure 2:**
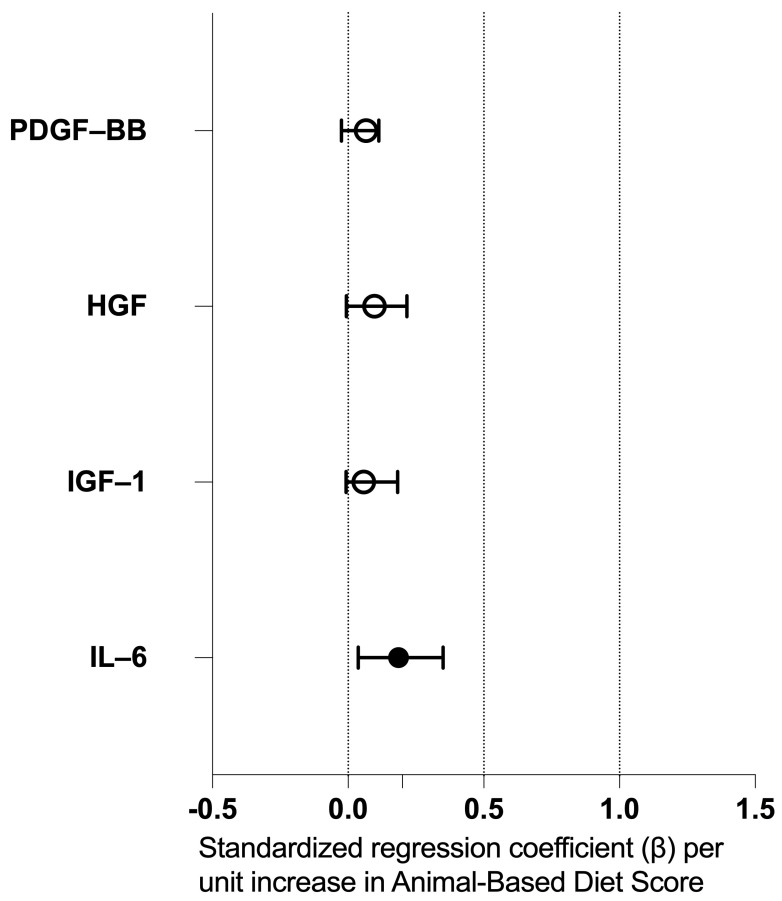
Standardized regression coefficients (β) for the association of the Animal-Based Diet Score with log_10_-transformed PRP protein concentrations (IL-6, IGF-1, HGF, and PDGF-BB) as dependent variables in multivariable models adjusted for age, sex, and BMI (n = 68); 95% confidence intervals derived from the unstandardized regression coefficients. Filled points indicate *p* < 0.05; open points indicate *p* ≥ 0.05.

In the four multivariable regression models adjusted for age, sex, and BMI, the Animal-Based Diet Score was positively associated with log_10_-transformed IL-6 concentrations. A higher Animal-Based Diet Score predicted higher IL-6 levels (standardized β = 0.35; unstandardized B = 0.112, 95% CI 0.037–0.186; *p* = 0.004). For IGF-1, no significant associations with the Animal-Based Diet Score were observed (β = 0.18; B = 0.025, 95% CI −0.007–0.058; *p* = 0.126), whereas a higher age was independently linked to lower IGF-1 levels (β = −0.26; B = −0.024, 95% CI −0.046–−0.001; *p* = 0.039). For HGF, the Animal-Based Diet Score showed a positive trend (β = 0.22; B = 0.046, 95% CI −0.006–0.097; *p* = 0.080), but this association did not reach statistical significance. No significant associations were found for PDGF-BB (β = 0.11; B = 0.020, 95% CI −0.025–0.066; *p* = 0.373) (Figure 2).

## 4. Discussion

To the best of our knowledge, this study is among the first to systematically analyze the association between habitual dietary patterns and the cellular and molecular composition of LP-PRP. Three key findings emerged from this study: First, while systemic platelet and erythrocyte counts were comparable across dietary groups, circulating leukocytes—particularly lymphocytes and basophils—differed significantly, supporting the evidence for effects of diet on systemic blood cell profiles. However, these cellular differences did not translate into PRP, where no group-specific variations in cell composition or platelet enrichment ratio were observed. Second, growth factor concentrations (IGF-1, HGF, and PDGF-BB) did not differ between dietary groups, but platelet-growth factor coupling was strongest among omnivores, and growth factors correlated with the intake of dietary components. Third, IL-6 PRP levels were lower in vegans compared with omnivores, and the Animal-Based Diet Score independently predicted IL-6 in PRP after an adjustment for age, sex, and BMI. Taken together, our results indicate that a habitual diet is associated with selected molecular components of LP-PRP.

The regenerative and immunomodulatory potential of PRP, which is increasingly used in the treatment of musculoskeletal conditions and in particular for the therapy of OA, remains controversial, with compositional variability likely being a major factor that underlies inconsistent clinical outcomes. Identifying the determinants of this variability is essential to enhancing the reproducibility and interpretability of PRP research. In this context, elucidating how dietary behavior, which is known to affect systemic cellular and protein profile [13,14,15,16], is also associated with LP-PRP composition may help to better understand and contextualize donor-related PRP variability.

In a first step, we analyzed the association between dietary pattern (vegan, vegetarian, and omnivorous) and the systemic blood cell profile. Our findings are consistent with several points from the literature. Large cross-sectional and interventional studies show that diet influences systemic leukocyte populations, with plant-based eaters having lower total leukocyte counts [16,30]. Supporting these results, in our study, vegetarians had higher total leukocyte counts than vegans, and the lymphocyte proportions were highest in omnivores. However, a previous epidemiological analysis showed progressively higher leukocyte counts and their subsets from vegan through vegetarian to regular meat eaters [16]. Apart from positive correlations between whole blood lymphocytes and both the Animal-Based Diet Score and fish consumption, as well as an inverse correlation between vegetable intake and eosinophil proportions, we did not observe this gradient in direct group comparisons within our small, homogeneous cohort of healthy adults. This finding is in line with a recent analysis of more than 8000 participants, which also reported no significant differences in leukocyte concentrations between vegetarians and omnivores after adjustment [31]. Furthermore, we found no differences in platelet counts, which is consistent with previous findings [31]. Despite the observed associations between dietary groups and systemic leukocyte profiles, these associations did not translate into LP-PRP in this study—beyond platelet counts and enrichment ratios, leukocyte counts and their subtypes remained stable across dietary groups, and the correlation analysis revealed largely no effect of the dietary pattern on cell profiles in PRP. The observed lack of translation from whole blood associations to PRP is encouraging, as it suggests a robustness of LP-PRP cellular composition despite moderate systemic variations. However, this stability may also reflect the intrinsically low leukocyte content of LP-PRP. Future studies that include leukocyte-rich PRP preparations will be needed to determine whether higher leukocyte concentrations preserve systemic differences within the PRP product.

Beyond the effects on cell counts, recent studies have shown that dietary behavior can also influence whole blood platelet and leukocyte function [32,33]. For instance, a higher olive oil intake was associated, ex vivo, with reduced platelet function, while the platelet counts remained unchanged [34]. Therefore, as a second analytical step, we analyzed proteins in LP-PRP after in vitro-induced platelet lysis, as shown before [28].

The analyzed growth factors HGF, IGF-1, and PDGF-BB likewise showed no significant concentration differences in LP-PRP when directly comparing vegans, vegetarians, and omnivores, suggesting that the regenerative potential of LP-PRP that is mediated by these growth factors is not strongly associated with the dietary patterns examined. However, our study revealed a potential modulatory effect of dietary patterns on the associations between platelet counts and growth factors in PRP. While PDGF-BB exhibited a diet-independent positive correlation with platelet levels in LP-PRP, HGF and IGF-1 were only linked to platelet counts in the omnivore cohort. Moreover, PDGF-BB levels were positively correlated with a meat intake and inversely correlated with fruit and vegetable consumption, further suggesting possible dietary influences on PRP composition. However, this finding should be interpreted cautiously, given the exploratory nature of the analysis.

In addition to the growth factors, the biological activity of PRP is attributed in part to its cytokine content, particularly regarding its immunomodulatory potential. Notably, we observed significantly lower IL-6 levels in LP-PRP from vegans compared to omnivores. Furthermore, regression analyses adjusting for age, sex, and BMI—factors known to affect PRP composition [7,35]—revealed a positive association between IL-6 concentrations and the Animal-Based Diet Score. Given that detected IL-6 in PRP can be considered to originate predominantly from plasma and leucocytes rather than platelets [36,37], and considering the current evidence on the anti-inflammatory effects of plant-based diets, IL-6 findings from this study appear both plausible and biologically intriguing. The associations with IL-6 may reflect known dietary mechanisms: Saturated fatty acids and heme iron, which are abundant in meat, can activate inflammatory pathways in immune cells, thereby enhancing IL-6 release [38,39,40]. Conversely, plant-based diets are typically rich in anti-inflammatory micronutrients, which may counteract these processes [33]. Although our study was descriptive in nature, these findings may provide a potential biological rationale for the reduced IL-6 levels observed in LP-PRP among individuals with a low meat intake. Vegan and vegetarian diets are conceptually distinct; however, we did not observe statistically significant differences in LP-PRP protein concentrations, particularly IL-6, between these two groups. This is consistent with their dietary profiles: both vegans and vegetarians reported no intake of (processed) meat and fish, and similarly, high fruit and vegetable consumption, differing mainly in non-meat animal-derived foods, which were more frequently consumed by vegetarians and corresponded to only numerically higher, non-significant IL-6 levels. Together with the stepwise increase of the Animal-Based Diet Score and IL-6 from vegans to vegetarians to omnivores and the positive associations of IL-6 with both meat and non-meat animal-derived foods, these findings suggest that diet-related variations in LP-PRP IL-6 in our cohort are mainly driven by a higher meat intake in omnivores, with additional contributions from non-meat animal-derived foods along the vegan–vegetarian–omnivore gradient, rather than by the categorical distinction between vegan and vegetarian diets alone.

This study has several limitations that should be considered when interpreting the findings. Its cross-sectional design precludes causal inference, and all observed associations must be regarded as exploratory. Although the cohort of young and healthy adults minimized confounding from comorbidities, medication use, and systemic inflammation, this narrow demographic limits generalizability to older or clinically affected populations, such as patients with osteoarthritis, where PRP composition and activity may differ. Furthermore, dietary patterns were assessed through self-report using a validated FFQ, but adherence was not externally verified. Dietary exposure was derived from aggregated FFQ food-group frequency categories rather than detailed quantitative intake data, which may have limited the detection of subtle differences between dietary profiles. Although the internally developed Animal-Based Diet Score showed internal consistency, it has not yet undergone external validation; findings based on this score should, therefore, be interpreted cautiously. Variables such as micronutrient supplementation (e.g., vitamin B12, vitamin D, omega-3 fatty acids, etc.), environmental and occupational factors, and women’s hormonal status were not assessed and may have contributed to unexplained variability. Moreover, our molecular assessment was limited to a predefined protein panel, and future studies should evaluate a broader range of growth factors and pro- and anti-inflammatory cytokines. The IL-6 assay used in this study quantifies total IL-6 and, therefore, does not allow conclusions regarding the IL-6 signaling context or the distribution across free versus complexed forms of IL-6. In addition, fasting status and exact blood draw time were not systematically controlled or analyzed; however, previous work has shown that leukocyte-poor PRP composition remains stable across clinically relevant blood collection times, suggesting limited impact on LP-PRP composition in this context [26]. Finally, although the sample size was balanced across dietary groups, it limited statistical power to detect small effects or perform subgroup analyses, and only predefined primary analyses were adjusted for multiple testing. Exploratory correlations were uncorrected and should be considered hypothesis generating.

Future research should evaluate whether the present findings can be replicated in larger and more diverse cohorts, including disease-specific populations, where PRP cellular profiles and biological activity may differ. Controlled dietary intervention studies are needed to determine whether dietary modification can causally alter PRP composition and whether such changes translate into meaningful biological or clinical effects.

## 5. Conclusions

This exploratory, hypothesis-generating study indicates that habitual dietary patterns are associated with the molecular rather than the cellular composition of leukocyte-poor PRP. While systemic leukocyte profiles differed modestly between dietary groups, PRP cell counts and platelet enrichment ratios remained largely stable. In contrast, IL-6 concentrations in LP-PRP were lower in vegans compared to omnivores, and the Animal-Based Diet Score independently predicted IL-6 levels in PRP after an adjustment for age, sex, and BMI. Correlation patterns between platelet counts, growth factors, and dietary components further suggest that diet may influence PRP protein profiles in a nuanced, pathway-specific manner rather than through uniform shifts across dietary groups.

Given that the molecular signature is considered an important determinant of PRP function, these findings indicate that habitual diet may represent a potential pre-analytical factor that is associated with interindividual variability in PRP. However, all results should be regarded as preliminary. Nutritional preconditioning before PRP preparation remains a conceptual framework that requires confirmation in larger cohorts and targeted dietary intervention studies to determine whether diet can meaningfully modulate PRP composition.

## Figures and Tables

**Table 1:** Baseline demographic characteristics and FFQ-derived dietary intake frequencies stratified by dietary pattern.

	Total (*n* = 75)	Dietary Pattern			*p*-Value
		Vegan (*n* = 25)	Vegetarian (*n* = 25)	Omnivore (*n* = 25)	
Sex, *n* (%)					0.850
Female	42 (56%)	14 (56%)	15 (60%)	13 (52%)	
Male	33 (44%)	11 (44%)	10 (40%)	12 (48%)	
Age (years), mean ± SD; IQR	25.92 ± 4.17; 6.00	26.60 ± 4.52; 7.00	25.72 ± 3.62; 6.00	25.44 ± 4.39; 7.00	0.407
BMI (kg/m^2^), mean ± SD; IQR	22.56 ± 3.17; 3.94	23.06 ± 3.52; 3.93	22.86 ± 3.37; 4.83	21.76 ± 2.51; 2.41	0.414
Sport activity (hours per week), mean ± SD; IQR	5.75 ± 3.68; 5.25	6.07 ± 3.17; 4.50	6.62 ± 4.65; 5.75	4.43 ± 2.70; 5.38	0.216
Dietary Variables *					
(Processed) meat; median; IQR	1; 2	1; 0	1; 0	3.5; 1	<0.001
Non-meat animal-derived foods; median; IQR	4; 4	1; 0	4; 1	5; 1	<0.001
Fish; median; IQR	1; 1	1; 0	1; 0	2; 1	<0.001
Fruits and vegetables; median; IQR	6; 1	6; 1	6; 1	5; 2	<0.001
Animal-Based Diet Score median; IQR	5; 6	2; 0	5; 1	8; 2	<0.001

* Frequency categories for the dietary variables: 1 = never; 2 = ≤1×/month; 3 = 2–4×/month; 4 = 2–3×/week; 5 = 4×/week–daily; 6 = several times/day. The Animal-Based Diet Score reflects the frequency of consumption of meat and non-meat animal-derived foods; higher scores indicate higher frequency.

**Table 2:** Cell analysis in whole blood and LP-PRP stratified by dietary pattern.

		Dietary Pattern						*p*-Value
		Vegan		Vegetarian		Omnivore		
		Mean	SD	Mean	SD	Mean	SD	
Whole blood	Platelets (/nL)	225.56	52.86	241.16	59.33	231.96	55.12	0.646
	Erythrocytes (×10^3^/nL)	4.62	0.39	4.72	0.46	4.77	0.43	0.673
	Leukocytes (/nL)	5.35	1.49	6.43	1.93	5.78	1.11	0.024
	Lymphocytes (%)	30.38	6.43	30.31	7.36	33.80	5.91	0.039
	Monocytes (%)	6.10	1.36	5.81	1.30	5.56	1.53	0.432
	Basophils (%)	0.72	0.24	0.54	0.22	0.63	0.27	0.043
	Eosinophils (%)	2.44	2.03	2.76	2.55	2.94	1.72	0.148
	Neutrophils (%)	58.13	6.96	58.50	8.49	54.70	7.39	0.094
LP-PRP	Platelets (/nL)	457.68	119.17	493.92	131.32	496.76	147.39	0.497
	Erythrocytes (×10^3^/nL)	0.02	0.04	0.03	0.05	0.03	0.05	0.577
	Leukocytes (/nL)	0.06	0.04	0.06	0.04	0.07	0.05	0.807
	Lymphocytes (%)	28.96	23.63	29.74	22.50	27.17	22.43	0.906
	Monocytes (%)	11.84	6.21	10.63	5.95	10.18	8.14	0.537
	Basophils (%)	1.39	2.20	1.02	2.57	0.84	1.62	0.557
	Eosinophils (%)	5.80	3.68	6.05	3.92	7.17	3.41	0.324
	Neutrophils (%)	48.38	19.65	48.66	20.35	48.72	16.87	0.955
PRP-to-whole blood platelet ratio		2.05	0.34	2.06	0.33	2.14	0.39	0.846

**Table 3:** Correlations between platelet concentrations in whole blood and LP-PRP and protein levels in LP-PRP across dietary patterns.

		Platelets Whole Blood ^1^			Platelets PRP ^1^		
		Vegan	Vegetarian	Omnivore	Vegan	Vegetarian	Omnivore
IL-6	ρ *p*-Value	0.288 0.162	0.150 0.474	0.199 0.339	0.116 0.582	0.176 0.401	0.483 * 0.014
IGF-1	ρ *p*-Value	0.263 0.204	0.078 0.712	0.229 0.272	0.098 0.641	0.020 0.924	0.412 * 0.041
HGF	ρ *p*-Value	0.380 0.061	0.135 0.538	0.031 0.884	0.258 0.214	0.100 0.650	0.416 * 0.039
PDGF-BB	ρ *p*-Value	0.485 * 0.014	0.378 0.068	0.355 0.082	0.544 * 0.005	0.471 * 0.020	0.624 * <0.001

^1^ Correlations are shown for platelet concentrations (/nL) in whole blood and LP-PRP with protein concentrations (pg/mL) in LP-PRP. Spearman’s rank correlation coefficients (ρ) and corresponding *p*-values are reported for each dietary pattern. * *p* < 0.05 indicates statistical significance.

**Table 4:** Correlations between hematological parameters in LP-PRP and dietary variables.

		Dietary Variables ^1^				
		(Processed) Meat	Non-Meat Animal-Derived Foods	Fish	Fruits and Vegetables	Animal-Based Diet Score
Platelets (/nL)	ρ *p*-Value	−0.022 0.856	0.057 0.637	−0.019 0.878	−0.099 0.400	0.001 0.998
Erythrocytes (×10^3^/nL)	ρ *p*-Value	0.134 0.273	0.195 0.104	0.146 0.232	−0.187 0.111	0.141 0.251
Leukocytes (/nL)	ρ *p*-Value	0.150 0.218	0.255 * 0.032	0.219 0.071	−0.064 0.586	0.226 0.064
Lymphocytes (%)	ρ *p*-Value	−0.033 0.787	0.044 0.715	0.073 0.551	0.019 0.087	0.020 0.869
Monocytes (%)	ρ *p*-Value	−0.123 0.315	−0.148 0.219	−0.167 0.170	−0.146 0.215	−0.181 0.139
Basophils (%)	ρ *p*-Value	−0.047 0.703	−0.236 * 0.048	−0.173 0.154	0.255 0.054	−0.151 0.218
Eosinophils (%)	ρ *p*-Value	0.200 0.099	0.143 0.234	0.084 0.495	−0.212 0.070	−0.181 0.140
Neutrophils (%)	ρ *p*-Value	−0.042 0.731	−0.095 0.433	−0.103 0.402	0.142 0.228	−0.085 0.490

^1^ Correlations are shown for dietary variables and hematological parameters in LP-PRP. Spearman’s rank correlation coefficients (ρ) and corresponding *p*-values are reported. * *p* < 0.05 indicates statistical significance.

**Table 5:** Correlations between protein concentrations in LP-PRP and dietary variables.

		Dietary Variables ^1^				
		(Processed) Meat	Non-Meat Animal-Derived Foods	Fish	Fruits and Vegetables	Animal-Based Diet Score
IL-6	ρ *p*-Value	0.242 * 0.045	0.407 * <0.001	0.074 0.544	−0.078 0.509	0.356 * 0.003
IGF-1	ρ *p*-Value	0.093 0.448	0.191 0.111	0.053 0.663	−0.043 0.718	0.161 0.188
PDGF-BB	ρ *p*-Value	0.261 * 0.032	0.174 0.150	0.181 0.140	−0.340 * 0.003	0.232 0.059
HGF	ρ *p*-Value	0.023 0.851	0.227 0.061	−0.029 0.815	−0.101 0.399	0.124 0.323

^1^ Correlations are shown for dietary variables and protein concentrations (pg/mL) in LP-PRP. Spearman’s rank correlation coefficients (ρ) and corresponding *p*-values are reported. * *p* < 0.05 indicates statistical significance.

## Data Availability

The data presented in this study are available from the corresponding author upon reasonable request and subject to institutional and ethical approval. A preliminary subset of these data has been published previously as part of an exploratory analysis [41].

## Abbreviations

The following abbreviations are used in this manuscript:

ACR	American College of Rheumatology
ADAMTS	A disintegrin and metalloproteinase with thrombospondin motifs
ASA	Acetylsalicylic acid
BMI	Body mass index
CI	Confidence interval
DMARD	Disease-modifying anti-rheumatic drug
DMOAD	Disease-modifying osteoarthritis drug
EDTA	Ethylenediaminetetraacetic acid
ELISA	Enzyme-linked immunosorbent assay
ESSKA	European Society of Sports Traumatology, Knee Surgery and Arthroscopy
FFQ	Food-frequency questionnaire
HGF	Hepatocyte growth factor
IGF	Insulin-like growth factor
IL	Interleukin
IQR	Interquartile range
LPS	Lipopolysaccharide
LP-PRP	Leukocyte-poor platelet-rich plasma
LR-PRP	Leukocyte-rich platelet-rich plasma
mL	milliliter
MMP	Matrix metalloproteinase
nL	nanoliter
NSAIDs	Non-steroidal anti-inflammatory drugs
OA	Osteoarthritis
OARSI	Osteoarthritis Research Society International
PDGF	Platelet-derived growth factor
pg	Picogram
PRP	Platelet-rich plasma
rpm	Revolutions per minute
SD	Standard deviation
SPSS	Statistical Package for the Social Sciences
